# Informing the Borrowing Process for Dose‐Finding Trials by Estimating the Similarity Between Population‐Specific Dose‐Toxicity Curves

**DOI:** 10.1002/pst.70067

**Published:** 2025-12-19

**Authors:** Dario Zocholl, Heiko Götte, Christina Habermehl, Burak Kürsad Günhan

**Affiliations:** ^1^ Institute for Medical Biometry, Informatics and Epidemiology, Faculty of Medicine University of Bonn Bonn Germany; ^2^ Merck Healthcare KGaA Darmstadt Germany

**Keywords:** Bayesian borrowing, dose‐finding, meta‐analysis, pediatric

## Abstract

The conduct of dose‐finding trials can be specifically challenging in small populations, for example, in pediatric settings. Recently, research has shown that Bayesian borrowing from adult trials combined with appropriately robust prior distributions enables the conduct of pediatric dose‐finding trials with very small sample sizes. However, the appropriate degree of borrowing remains a subjective choice, relying on default methods or expert opinion. This paper proposes an approach to empirically determine the degree of borrowing based on a meta‐analysis of the similarity between population‐specific dose‐toxicity curves of other biologically similar compounds. Focusing on the pediatric use case, the approach may be applicable to any dose‐finding trial with information borrowing from another population. With the ExNex and a hierarchical model, two popular statistical modeling approaches are applied. The estimated degree of similarity is then translated into the statistical model for the dose‐finding algorithm using either variance inflation or robust mixture prior distributions. The performance of each combination of statistical model approaches is investigated in a simulation study. The results with mixture priors are promising for the application of the proposed methods, especially with many (20) compounds, while variance inflation models require additional fine‐tuning and seem to be less robust. With fewer (3 or 7) compounds, our proposed methods are either in line with robust priors that ignore the data from other compounds or are slightly better. We further provide a case study analyzing real dose‐finding data from 6 compounds with our models, demonstrating applicability in real‐world situations. For clinical trials teams, the decision for or against the proposed approach might be connected to the efforts in terms of time and cost to receive the external data.

## 1 Introduction

The conduct of dose‐finding trials can be specifically challenging in small populations, for example in pediatric settings. Such pediatric dose‐finding trials are rarely conducted, typically because of strong limitations to the sample size due to both feasibility and ethical concerns. Due to the lack of pediatric trials, off‐label use remains a major issue in pediatric health care [1, 2].

For dose‐finding trials in oncology, the Continual Reassessment Method (CRM [3, 4]) and its extensions, particularly the popular Bayesian Logistic Regression Model (BLRM [5]), have been established during the last decade as the statistically favorable alternative to easy‐to‐implement but less efficient rule‐based designs, such as the 3 + 3 design [6, 7].

The application of the CRM in the pediatric setting has been endorsed multiple times in the literature [8, 9]. To enhance trial performance, it seems natural to extrapolate information from adults to the pediatric trials, which is typically done within the Bayesian framework [10]. Several works have investigated methods to perform this extrapolation or borrowing of information within the framework of the CRM [11, 12, 13]. In simulation studies, it has been shown that pediatric dose‐finding trials with extremely small sample sizes are feasible under the application of robust informative priors that borrow information from adults [13].

An essential part of any borrowing approach is to determine an appropriate degree of borrowing. Robustification can make the algorithms less sensitive to prior‐data conflicts [13, 14], but the efficiency of these approaches is still naturally limited. The main idea of the current paper is that the degree of borrowing could be informed by an analysis of the similarity between dose‐toxicity curves of different populations in other biologically similar compounds. For many relevant target substances in oncology, multiple related substances with the same or a very similar mechanism are developed and investigated by competitors. For some of these, there may be trials already conducted in both relevant populations, so that information about their similarity could be derived, which could inform the degree of borrowing for the new trial in the target population.

We want to note that the motivation of this work was the application to pediatric dose‐finding trials when borrowing adult information. However, the methodology developed in the following is not restricted to this specific use case, but rather is applicable to any situation where information about dose‐toxicity curves is supposed to be borrowed from a *source* population to a *target* population. Another common use case may be the situation where information from one ethnicity should be borrowed in order to inform a dose‐finding trial in another ethnicity.

To share information across different populations in phase I trials, the use of hierarchical models has been proposed [15]. Recently, the application of a two‐stage meta‐analysis approach to dose‐finding trials has been suggested [16] to utilize all available information about a compound before initiating a dose‐finding trial. Although the quantification of study heterogeneity is an inherent component of this meta‐analysis approach, heterogeneity estimation for our purpose, i.e., to define the degree of borrowing, poses unresolved methodological challenges: it is important to acknowledge that our approach does not require estimating the heterogeneity of the dose‐toxicity curves across compounds, but the variability across compounds in how similar the dose‐toxicity curves are between source and target populations within each compound. Another study proposed distance measures to assess the similarity of two dose‐toxicity curves [17], but methods to estimate similarity using multiple compounds and to formally determine the degree of borrowing are still lacking.

Our aim in this paper is twofold: first, we demonstrate how the degree of borrowing between populations in a dose‐finding trial can be determined and second, we investigate whether this would be potentially useful for practical application.

In Section 2, we outline the CRM methodology and borrowing methods used in this paper. In Section 3, we propose methods to estimate the degree of similarity between population‐specific dose‐toxicity curves from multiple compounds, and show how the estimated similarity can be incorporated into the existing borrowing framework. In Section 4, we describe the design of a simulation study to evaluate the performance of our methods, the results of which are presented in Section 5. A case study is provided in Section 6. The implications of our results are discussed in Section 7.

## 2 Materials and Methods

To identify the maximum tolerated dose, the Continual Reassessment Method (CRM) provides an adaptive framework. The maximum tolerated dose is the dose with a prespecified target probability θ of a dose‐limiting toxicity (DLT). The number of patients with DLT per dose level $k\in \left\{1,\ldots ,K\right\}$ follows a binomial distribution with $Y_{k}∼\mathrm{Bin}\left(n_{k},p_{k}\right)$, where $n_{k}$ is the number of patients treated on dose level k and $p_{k}$ the corresponding probability of a DLT. After a cohort of patients has been treated and their outcomes have been observed, the dose for the next cohort is determined as the dose with an estimated probability of DLT closest to θ. Various models to estimate $p_{k}$ have been introduced, but in the recent literature, the two‐parameter Bayesian logistic regression model (BLRM) has become the predominant method and we will use it here, too.

### 2.1 The Bayesian Logistic Regression Model (BLRM) for Dose‐Finding

The two‐parameter logistic model is given by:

$$\text{logit}\;p\left(D;\alpha ,\beta \right)=\log\left(\alpha \right)+\beta \log\left(D/D_{R}\right),\text{for}\;\alpha >0,\beta >0,$$

where D is the dose of interest, and $D_{R}$ is a reference dose. We assign bivariate normal priors for $\log\left(\alpha \right)$ and $\log\left(\beta \right)$ with means $\mu_{\alpha }$ and $\mu_{\beta }$, respectively, variances $\sigma_{\alpha }^{2}$ and $\sigma_{\beta }^{2}$, and covariance $\sigma_{\alpha ,\beta }$:

$$\left(\begin{array}{c} \log\left(\alpha \right)\\ \log\left(\beta \right) \end{array}\right)∼N\left(\left(\begin{array}{c} \mu_{\alpha }\\ \mu_{\beta } \end{array}\right),\left(\begin{array}{cc} \sigma_{\alpha }^{2} & \sigma_{\alpha ,\beta }\\ \sigma_{\alpha ,\beta } & \sigma_{\beta }^{2} \end{array}\right)\right).$$

The dosing decisions are based on the posterior distributions of the model parameters, which are obtained by employing Markov Chain Monte Carlo methods, for which we will employ implementations in JAGS [18] and Stan [19].

To define weakly informative priors, multiple ways have been proposed. Neuenschwander et al. [5] suggested to define weakly informative distributions on the toxicity probability scale using unimodal Beta distributions and approximating bivariate normal priors by optimization or to define weakly informative priors in terms of the intercept and slope directly, i.e., to define an a priori plausible range for the toxicity probability of the reference dose and for the change in the odds of a toxicity when the dose is changed. Based on the latter approach, we used $\sigma_{\alpha }=2$, $\sigma_{\alpha ,\beta }=0$, and $\sigma_{\beta }=1.5$, which allows for a relatively wide range of toxicity probabilities for both the reference dose as well as a change of dose and which is similar to values used elsewhere in the dose‐finding literature [13, 20]. Further motivation for these priors is provided in the Supporting Information.

### 2.2 Borrowing From a Source Population in Dose‐Finding Trials

If two trials use the same reference dose $D_{R}$, full borrowing can be achieved by using the posterior distribution of the preceding trial in the source population as prior distribution for the current trial in the target population. However, the posterior distribution of this model does not have a closed form. A simple approximation with a bivariate normal distribution with means $\mu_{\alpha ,\text{source}}$ and $\mu_{\beta ,\text{source}}$, variances $\sigma_{\alpha ,\text{source}}^{2}$, $\sigma_{\beta ,\text{source}}^{2}$ and covariance $\sigma_{\alpha ,\beta ,\text{source}}$ is sufficient in many situations [13].

Full borrowing can be problematic, particularly in these small samples, if the prior dominates the data. Then, the dosing decision is mainly based on the prior information from the source population instead of the relevant information about the target population. Robust priors are priors that are specified such that they give more weight to the data in case of prior‐data conflicts. A common approach to achieve robust priors is partial borrowing, for which we will introduce two versions in the following.

#### 2.2.1 Controlling the Degree of Borrowing via Robust Mixture Priors

The first approach to control the degree of borrowing that we use in this paper is by employing robust mixtures of prior distributions [13], where a weakly informative component and one component with full borrowing are combined with a weighting parameter δ:

$$\left(\begin{array}{c} \log\left(\alpha \right)\\ \log\left(\beta \right) \end{array}\right)∼\delta \times P_{\text{source}}\left(\log\left(\alpha \right),\log\left(\beta \right)\right)+\left(1-\delta \right)\times P_{\text{weak}}\left(\log\left(\alpha \right),\log\left(\beta \right)\right)$$

The closer δ is to 0 the less informative the prior, δ=1 means full borrowing. It is possible to use a prespecified fixed mixture weight, e.g., equal weighting

δ=0.5.

It may also be desirable to borrow only information about the slope or only information about the intercept from the source data. For this, two distinct mixture parameters for each parameter, i.e., $\delta_{\alpha }$ and $\delta_{\beta }$, may be considered. However, this requires to treat α and β as independent:

$$\begin{array}{l} \log\left(\alpha \right)∼\delta_{\alpha }\times P_{\text{source}}\left(\log\left(\alpha \right)\right)+\left(1-\delta_{\alpha }\right)\times P_{\text{weak}}\left(\log\left(\alpha \right)\right)\\ \log\left(\beta \right)∼\delta_{\beta }\times P_{\text{source}}\left(\log\left(\beta \right)\right)+\left(1-\delta_{\beta }\right)\times P_{\text{weak}}\left(\log\left(\beta \right)\right) \end{array}$$

In our simulations, the impact of the prior covariance of the model parameters appeared to have little influence on the posterior distribution, in contrast to the flexibility to define various degrees of borrowing for the model parameters. Therefore, in the following, we consider the latter, independent formulation for the robust mixture priors.

#### 2.2.2 Controlling the Degree of Borrowing via Variance Inflation

Analogously to the similarity parameter δ in the mixture priors, a variance inflation parameter ω can be used to inflate the variance of an informative prior in case of a prior‐data conflict. For the BLRM, this has been proposed by Takeda and Morita (2018) [12]:

$$\left(\begin{array}{c} \log\left(\alpha \right)\\ \log\left(\beta \right) \end{array}\right)∼N\left(\left(\begin{array}{c} \mu_{\alpha ,\text{source}}\\ \mu_{\beta ,\text{source}} \end{array}\right),\left(\begin{array}{cc} \frac{\sigma_{\alpha ,\text{source}}^{2}}{\omega } & \sigma_{\alpha ,\beta ,\mathrm{adj}}\\ \sigma_{\alpha ,\beta ,\mathrm{adj}} & \frac{\sigma_{\beta ,\text{source}}^{2}}{\omega } \end{array}\right)\right),$$

where $\sigma_{\alpha ,\beta ,\mathrm{adj}}=\rho_{\text{source}}\times \sqrt{\frac{\sigma_{\alpha ,\text{source}}^{2}}{\omega }}\times \sqrt{\frac{\sigma_{\beta ,\text{source}}^{2}}{\omega }}$ with $\rho_{\text{source}}$ being the correlation between the parameters in the posterior distribution of the trial in the source population. The closer ω is to 0 the less informative the prior, a value of 1 means full borrowing. The parameters can be fixed, e.g., to 0.5, or estimated. It is possible to use a prespecified fixed inflation parameter, e.g.,

ω=0.5.

In contrast to the robust mixture approach, it is straightforward to define distinct similarity parameters for α and β, respectively, in the bivariate model:

$$\left(\begin{array}{c} \log\left(\alpha \right)\\ \log\left(\beta \right) \end{array}\right)∼N\left(\left(\begin{array}{c} \mu_{\alpha ,\text{source}}\\ \mu_{\beta ,\text{source}} \end{array}\right),\left(\begin{array}{cc} \frac{\sigma_{\alpha ,\text{source}}^{2}}{\omega_{\alpha }} & \sigma_{\alpha ,\beta ,\mathrm{adj}}\\ \sigma_{\alpha ,\beta ,\mathrm{adj}} & \frac{\sigma_{\beta ,\text{source}}^{2}}{\omega_{\beta }} \end{array}\right)\right),$$

where $\sigma_{\alpha ,\beta ,\mathrm{adj}}=\rho_{\text{source}}\times \sqrt{\frac{\sigma_{\alpha ,\text{source}}^{2}}{\omega_{\alpha }}}\times \sqrt{\frac{\sigma_{\beta ,\text{source}}^{2}}{\omega_{\beta }}}$ with $\rho_{\text{source}}$ being the correlation between the parameters in the posterior distribution of the trial in the source population.

## 3 New Approach: Estimating Similarity to Define the Degree of Borrowing

While several methods exist to control the degree of borrowing using some kind of weighting parameter, the question remains how a specific choice for the weighting parameter can be decided upon. Since the sample size is very limited, an internal dynamic borrowing approach which estimates the similarity between the new data and the information borrowed from the other population ‘on the go’ during the trial is not feasible. Instead, we propose to estimate the similarity from data of other compounds that have a similar mechanism of action as the target compound.

**FIGURE 1:**
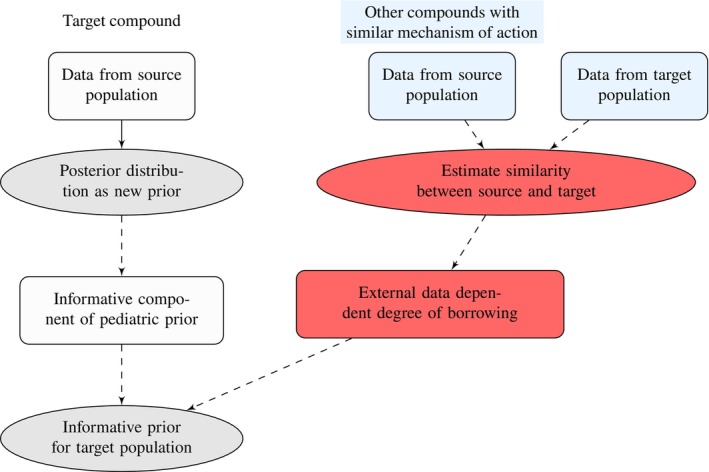
A visualization of the proposed approach. The steps marked in red indicate the novel contribution of this work.

It is crucial to distinguish two components of the information borrowing process. The first is the actual information to be borrowed. In our approach, this is the information from a preceding trial in the source population with the *same* compound. The second component is the degree of borrowing of this information, which we propose to estimate from *other* compounds. Our approach therefore does not contain borrowing of information about the dose‐toxicity curves from other compounds, and thus does not make any assumptions about the similarity of dose‐toxicity curves across compounds. The only assumption is that the average similarity between source and target dose‐toxicity curves from other compounds can also be assumed for the target compound to be tested. In the visualization of our approach (Figure 1), this is represented by the two separate workflows for the target compound and other compounds.

In the following, we present two novel model approaches which can be used to estimate the similarity between adult and pediatric dose‐toxicity curves. Both approaches aim to estimate similarity parameter $\zeta_{\alpha }$ and $\zeta_{\beta }$, which will be used to determine values for $\delta_{\alpha }$ and $\delta_{\beta }$ in the robust mixture approach and $\omega_{\alpha }$ and $\omega_{\beta }$ in variance inflation model, respectively, to make an informed decision about the degree of borrowing.

### 3.1 ExNex Model

To decide whether multiple strata are exchangeable or not, the ExNex approach [21] has been proposed. At its core, the ExNex approach models the exchangeability of strata with a robust mixture distribution that contains an exchangeable (Ex) and a non‐exchangeable (Nex) component. Depending on the prior and the data, one of the two components is assigned more weight.

For our situation of j compounds, we assume compound‐specific exchangeability of source (S) and target (T) dose‐toxicity curves, but we do not assume exchangeability of dose‐toxicity curves between compounds. To this end, we build the ExNex model in the following way.

**Exchangeable component (Ex)** With probabilities $\zeta_{\alpha }$ and $\zeta_{\beta }$, the parameters for source and target dose‐toxicity curves come from the same compound‐specific distribution, i.e.,:

$$\begin{array}{l} \alpha_{T,j},\alpha_{S,j}∼N\left(\mu_{\alpha ,j},\sigma_{\alpha }^{2}\right)\\ \beta_{T,j},\beta_{S,j}∼N\left(\mu_{\beta ,j},\sigma_{\beta }^{2}\right) \end{array}$$

**Non‐exchangeable component (Nex)** With probability $1-\zeta_{\alpha }$ and $1-\zeta_{\beta }$, the parameters for source and target dose‐toxicity curves come from different compound‐specific distributions, i.e.,:

$$\begin{array}{l} \alpha_{T,j}∼N\left(\mu_{\alpha_{T,j}},\sigma_{\alpha }^{2}\right),\alpha_{S,j}∼N\left(\mu_{\alpha_{S,j}},\sigma_{\alpha }^{2}\right)\\ \beta_{T,j}∼N\left(\mu_{\beta_{T,j}},\sigma_{\beta }^{2}\right),\beta_{S,j}∼N\left(\mu_{\beta_{S,j}},\sigma_{\beta }^{2}\right) \end{array}$$

Each parameter is modeled as a mixture between Ex and Nex; for the weights, a Beta prior is used:

$$\zeta_{\alpha }∼\text{Beta}\left(0.5,0.5\right)\;\text{and}\;\zeta_{\beta }∼\text{Beta}\left(0.5,0.5\right)$$

The variance parameters are assigned vague Gamma priors, $\Gamma \left(1,1/10\right)$. Motivation for these priors is provided in the Supporting Information. Provided some data, the posterior distributions of $\zeta_{\alpha }$ and $\zeta_{\beta }$ can then be obtained via MCMC methods.

### 3.2 Hierarchical Model

Another common approach to consider exchangeability are hierarchical models. As in the ExNex‐approach, we model the compound‐specific exchangeability of target and source population dose‐toxicity curves.

So, the first level of our hierarchical model are population‐specific and compound‐specific means, where the variance $\tau^{2}$ expresses population heterogeneity (within‐compound‐variability):

$$\left(\begin{array}{c} \log\left(\alpha_{S,j}\right)\\ \log\left(\beta_{S,j}\right) \end{array}\right),\left(\begin{array}{l} \log\left(\alpha_{T,j}\right)\\ \log\left(\beta_{T,j}\right) \end{array}\right)∼N\left(\left(\begin{array}{l} \mu_{\alpha_{j}}\\ \mu_{\beta_{j}} \end{array}\right),\left(\begin{array}{cc} \tau_{\alpha }^{2} & \tau_{\alpha ,\beta }\\ \tau_{\alpha ,\beta } & \tau_{\beta }^{2} \end{array}\right)\right)$$

To obtain a quantification of the degree of exchangeability in the hierarchical modeling framework, we additionally model the between‐compound variability in the second level, which is the distribution of compound‐specific means:

$$\left(\begin{array}{l} \mu_{\alpha_{j}}\\ \mu_{\beta_{j}} \end{array}\right)∼N\left(\left(\begin{array}{l} \mu_{\alpha }\\ \mu_{\beta } \end{array}\right),\left(\begin{array}{cc} \sigma_{\alpha }^{2} & \sigma_{\alpha ,\beta }\\ \sigma_{\alpha ,\beta } & \sigma_{\beta }^{2} \end{array}\right)\right)$$

with some rather weakly priors, for which we specify $\mu_{\alpha }∼N\left(-{0.84,2}^{2}\right)$, $\mu_{\beta }∼N\left({0,1.5}^{2}\right)$ and $\tau_{\alpha }∼\Gamma \left(1,1/10\right)$, $\tau_{\beta }∼\Gamma \left(1,1/10\right)$, $\rho_{\tau }∼\text{Unif}\left(-1,1\right)$, $\sigma_{\alpha }∼\Gamma \left(1,1/10\right)$, $\sigma_{\beta }∼\Gamma \left(1,1/10\right)$, $\rho_{\sigma }∼\text{Unif}\left(-1,1\right)$. Motivation for these priors is provided in the Supporting Information.

The similarity (or heterogeneity) parameters are then estimated as standard intraclass correlation coefficients:

$$\zeta_{\alpha }=\frac{\tau_{\alpha }^{2}}{\tau_{\alpha }^{2}+\sigma_{\alpha }^{2}}\;\text{and}\;\zeta_{\beta }=\frac{\tau_{\beta }^{2}}{\tau_{\beta }^{2}+\sigma_{\beta }^{2}}$$

### 3.3 Applying the Estimated Similarity to the Pediatric Prior Distribution

As the final step in the proposed workflow to define an informative prior distribution for the target population, the posterior similarity parameters $\zeta_{\alpha }$ and $\zeta_{\beta }$ need to be transferred to the target population prior. We propose to use the means of the posterior distributions, ${\hat{\zeta }}_{\alpha }$ and ${\hat{\zeta }}_{\beta }$. If the degree of borrowing is controlled via robust mixture priors, the weights of the informative components $\delta_{\alpha }$ and $\delta_{\beta }$ can simply be set to ${\hat{\zeta }}_{\alpha }$ and ${\hat{\zeta }}_{\beta }$, respectively. For the model with variance inflation, our simulations showed that in the considered scenarios the inflation often was not strong enough, so we applied a transformation to these parameters: $\omega_{\alpha }=\frac{0.01^{-{\hat{\zeta }}_{\alpha }}}{100}$ and $\omega_{\beta }=\frac{0.01^{-{\hat{\zeta }}_{\beta }}}{100}$. For example, assume a typical posterior variance of $\sigma_{\alpha ,\text{adults}}^{2}=0.3$ based on data from 40 adults, this transformation maps three possible values for $\zeta_{\alpha }=\left\{0.1,0.5,0.9\right\}$ (corresponding to strong evidence against similarity, no tendency, and strong evidence supporting similarity) to $\omega_{\alpha }=\left\{0.02,0.10,0.63\right\}$, which results in pediatric prior variance $\frac{\sigma_{\alpha ,\text{adults}}^{2}}{\omega_{\alpha }}=\left\{18.93,3.00,0.48\right\}$. Without the transformation, the pediatric prior variance would be $\frac{\sigma_{\alpha ,\text{adults}}^{2}}{\omega_{\alpha }}=\left\{3.0,0.6,0.33\right\}$, which is quite informative, particularly for the middle case of ‘no tendency’. In general, the appropriate transformation depends on the prior variance in the specific case, i.e., how much information is borrowed, which is a disadvantage in terms of generalizability of the method.

## 4 Design of Simulation Study

### 4.1 Simulation of the Historical Compounds With Adult and Pediatric Data

The first step of our proposed workflow consists of estimating the similarity parameters from the available data from other compounds. The following parameters of the data‐generating process are varied in the simulation:

- Four similarity scenarios:
  ◦ equal α and equal β
  ◦ equal α, β different
  ◦ different α, equal β
  ◦ different α and different β
- Two values each for $\log\left(\alpha \right)\in \left\{-2.5,-0.84\right\}$ and for $\log\beta \in \left\{0,1.5\right\}$, thus 4 × 4 dose‐toxicity curves.
- 3 vs. 7 vs. 20 compounds
- Sample size of 30 vs. 100 patients per trial

For simplicity of the simulation setup, all trials test the same 5 doses and there is an equal distribution of the sample size to the dose levels. However, this is by no means a necessary prerequisite for our methods to work. The purpose of this simplification is only to reduce variability in the simulation results and to improve interpretability. Within the four similarity scenarios, the two parameters α and β can take one of two values, which makes a total of four combinations. An additional data‐generating mechanism with random instead of fixed dose‐toxicity parameters is presented in the Supporting Information. For the simulation results of the dose‐finding in the next section, the differences among them will be relevant, but for the estimation of the similarity parameters each compound is randomly assigned one of the four combinations within each similarity scenario.

For each data‐generating process, 1000 simulation runs are performed. The main outcome of this simulation study is the posterior means of the similarity parameters, which should ideally be zero for scenarios with different parameters and 1 in case of scenarios with equal parameters.

### 4.2 Simulation of the New Pediatric Trial

In the second step of our proposed workflow, the estimated similarity parameters are transferred to the design of a new pediatric dose‐finding trial. We consider a setting where an adult trial has been conducted with *N* = 40, and we want to test the same compound in *N* = 12 pediatric patients in cohort sizes of 2 patients. For the estimation of the similarity parameter, we assume:

- an ideal case of 20 historical compounds with 100 pediatric and adult patients each,
- a more realistic situation with 7 historical compounds with 30 pediatric and adult patients each.

Note that the scenario with only 3 compounds from the previous simulation is not continued here because the results indicated that not much can be gained compared to a default weight, which is explained in more depth in the next section.

We further assume that the similarity between pediatric and adult dose‐toxicity curves is the same for the new compound as for the historical compounds. The following model approaches are considered:

- Weakly informative priors borrow posterior means but not posterior variance from the adults.
- Models with ‘full borrowing’ borrow posterior means and posterior variances from adults.
- The weights for mixture priors and the variance inflation parameters are determined by one the following strategies:
  ◦ Fixed at 0.5,
  ◦ Estimated from historical data with 20 vs. 7 cohorts using the ExNex model.
  ◦ Estimated from historical data with 20 vs. 7 cohorts using the hierarchical model.

Each approach is simulated 1000 times. Here, the main outcome is the accuracy of the new pediatric trials, i.e., the proportion of simulated trials that correctly identify the MTD.

The simulation code as well as the code for the Bayesian models in Stan [19] and JAGS [18] is provided at https://github.com/dariozchl/Manuscript‐informative‐pediatric‐dose‐finding.

## 5 Results of Simulation Study

### 5.1 Estimation of Similarity Parameters

**FIGURE 2:**
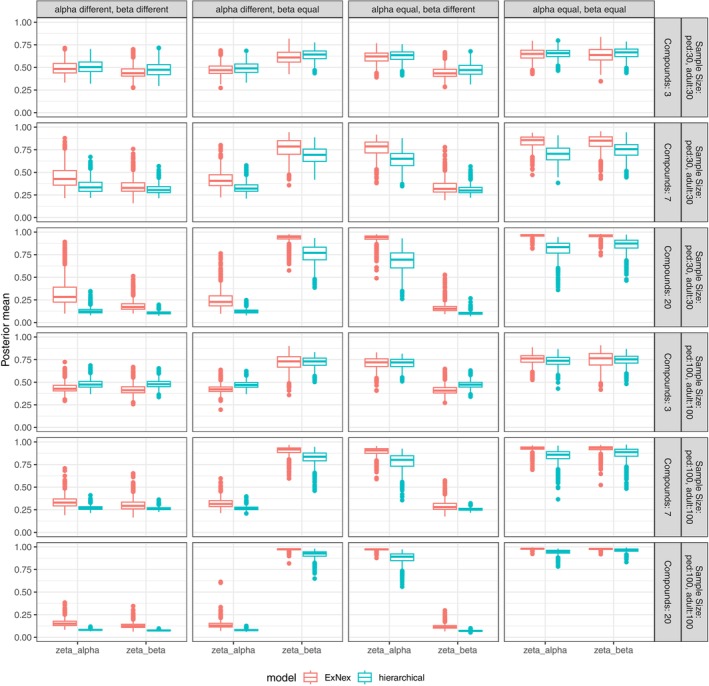
Simulation results: Posterior means of the estimated similarity parameters.

As described above, for the estimation of the similarity parameters, only the four similarity scenarios are relevant, while the distinction of the four dose‐toxicity curves within each similarity scenario will become important in the next chapter. Therefore, the simulation results can be displayed in a compact way, where the simulated posterior means are summarized in boxplots for each parameter, model, similarity scenario, and available data assumption (Figure 2). With only three compounds available with a sample size of 30 each, the posterior means barely deviate from the prior means of 0.5 in all scenarios and with both models. With more compounds and more sample size, respectively, the estimation becomes better: if the parameters of adult and pediatric dose‐toxicity curves differ, similarity parameters are estimated closer to 0, and if they are the same, posterior means tend toward 1. This observation is very similar for both ExNex and the hierarchical model. However, the hierarchical model rather provides estimates very close to 1 in case of identical parameters, while the ExNex model rather estimates posterior means very close to 0 in case of different parameters. The estimation seems to work very well with both models in the case of 20 compounds with a sample size of 100 each, with posterior means reaching values close to 0 and 1 in the respective cases with relatively little variability.

For the purpose of the further investigation of our proposed approach, we proceed with only two of these scenarios: the case with 7 compounds and a sample size of 30 each, and the case with 20 compounds and a sample size of 100 each. The first represents a rather realistic scenario, while the latter represents an ‘optimal benchmark’, which is probably unrealistic to appear in practice but is useful to explore the potential of the method. Since with just 3 compounds, the posterior means deviate only slightly from the prior means of 0.5, not much can be gained compared to a default weight of 0.5, so we discard this scenario from the further simulations in order to reduce the dimensionality and improve the interpretability of the simulation results.

### 5.2 Dose‐Finding With Informative Priors

**FIGURE 3:**
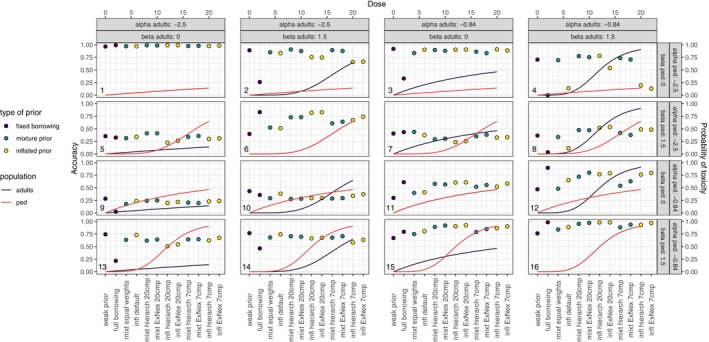
Simulation results based on 10,000 simulations per scenario: Accuracy, i.e., proportion of correctly identified MTD. The simulation results are displayed alongside of the adult and pediatric dose‐toxicity curve of the corresponding simulation scenario. For the dose‐toxicity curves, the *x*‐axis represents the dose and the *y*‐axis the probability of toxicity. For the simulation results, the *x*‐axis shows the different approaches and the *y*‐axis displays the accuracy, i.e., the proportion of correctly identified MTDs. The α and β parameters of the data‐generating dose‐toxicity curves can be read from the gray boxes on top (adult) and on the right (pediatric) of the figure. The numbers in the bottom left corners correspond to the simulation scenario numbering in Section 5.2.

The simulation results for the outcome accuracy, i.e., the percentage of correctly identified MTDs, are displayed in Figure 3. These results will be briefly described separately for each simulation scenario because it is important to take the individual interaction between pediatric and adult dose‐toxicity curve into account and whether the adult MTD, ${\mathrm{MTD}}_{A}$, is higher, lower or equal to the pediatric MTD, ${\mathrm{MTD}}_{P}$. The core characteristics of the scenario are provided at the beginning of each summary. A brief conclusion is provided at the end of the section.

1. $\alpha_{A}=\alpha_{P}$, $\beta_{A}=\beta_{P}$, ${\mathrm{MTD}}_{A}={\mathrm{MTD}}_{P}$. Pediatric and adult toxicity are identical. Toxicity is very low, so all configurations escalate quickly and achieve close to perfect accuracy. Even the weakly informative prior escalates fast, because it borrows the posterior means from the adult toxicity curve, which indicates low toxicity initial to the pediatric trial.
2. $\alpha_{A}=\alpha_{P}$, $\beta_{A}>\beta_{P}$, ${\mathrm{MTD}}_{A}<{\mathrm{MTD}}_{P}$. Intercepts are identical, slopes are different. Pediatric MTD is higher than adult MTD. With full borrowing, escalation in pediatric patients is too slow resulting in low accuracy. Mixture priors with informed weighting have almost perfect performance, closely followed by weakly informative priors as well as mixture priors and inflated variance priors with default weights. The models with informed inflated prior variance perform slightly worse, with information from 20 compounds still somewhat better than with 7 compounds.
3. $\alpha_{A}>\alpha_{P}$, $\beta_{A}=\beta_{P}$, ${\mathrm{MTD}}_{A}<{\mathrm{MTD}}_{P}$. Intercepts are different, slopes are identical. Pediatric MTD is higher than adult MTD. Except for full borrowing, which has rather poor accuracy similar to scenario 2, all configurations have very good accuracy around 90%.
4. $\alpha_{A}>\alpha_{P}$, $\beta_{A}>\beta_{P}$, ${\mathrm{MTD}}_{A}<{\mathrm{MTD}}_{P}$. Intercepts and slopes are different. Pediatric MTD is higher than adult MTD. For high doses, adult toxicity is much higher than pediatric toxicity, so strongly informative priors prevent necessary escalation in pediatric patients. Weakly informative and mixture priors perform similarly well. Inflated priors show good performance only with the hierarchical model and 20 compounds, but the ExNex approach and particularly the inflated variance models with 7 compounds and default weighting show lower accuracy due to insufficient variance inflation. Notably, weakly informative priors perform worse than in scenario 1 due to prior means being informed by the adult posterior means.
  This scenario makes a strong case for the lack of robustness of the inflated prior in specific situations and demonstrates the advantages of the mixture prior models.
5. $\alpha_{A}=\alpha_{P}$, $\beta_{A}<\beta_{P}$, ${\mathrm{MTD}}_{A}>{\mathrm{MTD}}_{P}$. Intercepts are the same, slopes are different. Pediatric MTD is lower than adult MTD. The correct information about the intercept is barely helpful, since the toxicity of the reference dose is quite small, thus providing little information about the correct MTD. The best performance is shown by mixture priors informed either by ExNex or by hierarchical model based on 20 compounds, borrowing intercept information but discarding slope, allowing fast escalation beyond the reference dose and careful exploration of higher doses. Other configurations show comparable accuracy; full borrowing tends to choose overly toxic doses as MTD, while weakly informative priors select too low doses.
6. $\alpha_{A}=\alpha_{P}$, $\beta_{A}=\beta_{P}$, ${\mathrm{MTD}}_{A}={\mathrm{MTD}}_{P}$. The intercepts and the slopes are identical. Full borrowing and inflated priors based on 20 compounds perform best, followed by mixtures informed by 20 compounds. Weakly informative priors have much lower accuracy than the informative configurations.
7. $\alpha_{A}>\alpha_{P}$, $\beta_{A}<\beta_{P}$, ${\mathrm{MTD}}_{A}={\mathrm{MTD}}_{P}$. The intercepts and the slopes are different, but adult and pediatric MTD are identical because the dose‐toxicity curves are crossing in the area of the MTD. The configurations with estimated similarity parameters perform worst, which may appear counter‐intuitive in light of the different dose‐toxicity curves, but is due to adult and pediatric MTD being identical.
8. $\alpha_{A}>\alpha_{P}$, $\beta_{A}=\beta_{P}$, ${\mathrm{MTD}}_{A}<{\mathrm{MTD}}_{P}$. The intercepts are different and the slopes are identical. Pediatric MTD is higher than the adult MTD. Inflated priors informed by 20 compounds, either by hierarchical model or ExNex, have the highest accuracy, followed by the informed mixture priors. Full borrowing performs very poor with accuracy close to 0, because the intercept is at the adult MTD. Accuracy of inflated variance priors with default weighting is similarly low, probably due to insufficient variance inflation.
9. $\alpha_{A}<\alpha_{P}$, $\beta_{A}=\beta_{P}$, ${\mathrm{MTD}}_{A}>{\mathrm{MTD}}_{P}$. Intercepts are different, slopes are identical. Pediatric MTD is lower than adult MTD. All specifications heavily struggle with this scenario, but full borrowing is by far the worst. Weakly informative priors perform best, closely followed by first the mixture prior with weight based on the hierarchical model with 20 compounds, and then rather closely by the other partial borrowing approaches.
10. $\alpha_{A}<\alpha_{P}$, $\beta_{A}>\beta_{P}$, ${\mathrm{MTD}}_{A}={\mathrm{MTD}}_{P}$. Intercepts and slopes are different, but adult and pediatric MTD are identical—as in scenario 7. And as in that scenario, the configurations with estimated similarity parameters perform slightly worse than the ones without informed borrowing.
11. $\alpha_{A}=\alpha_{P}$, $\beta_{A}=\beta_{P}$, ${\mathrm{MTD}}_{A}={\mathrm{MTD}}_{P}$. Intercepts and slopes are identical. Full borrowing performs best, both mixture and inflated priors come very close when informed by 20 compounds, both by ExNex and the hierarchical model. Weakly informative priors perform worst, and the borrowing approaches with default weights are only slightly better.
12. $\alpha_{A}=\alpha_{P}$, $\beta_{A}>\beta_{P}$, ${\mathrm{MTD}}_{A}={\mathrm{MTD}}_{P}$. The intercepts are identical and the slopes are different. The pediatric MTD and adult MTD are identical. Interestingly, the strongly informative priors performs best. This is because the intercept is at the true MTD, and the large adult slope indicates that all other doses are either too high or too low, which is not correct but still helpful because it supports fast escalation to and remaining at the true MTD. The partial approaches follow, mixture priors and inflated variance priors performing similarly better with 20 compounds than with 7 compounds. For both models, weights estimated by the ExNex approach perform better than weights estimated by the hierarchical model.
13. $\alpha_{A}<\alpha_{P}$, $\beta_{A}<\beta_{P}$, ${\mathrm{MTD}}_{A}>{\mathrm{MTD}}_{P}$. Intercepts and slopes are different. Pediatric MTD is lower than adult MTD. Weakly informative priors show the best accuracy, the approaches with partial borrowing come close, whereby the mixture priors perform slightly better than the inflated priors, except for default weights, and the number of available compounds for the estimation of the similarity does not appear to make a difference. Full borrowing has much lower accuracy than all other configurations.
14. $\alpha_{A}<\alpha_{P}$, $\beta_{A}=\beta_{P}$, ${\mathrm{MTD}}_{A}>{\mathrm{MTD}}_{P}$. The intercepts are different and the slopes are identical. The pediatric MTD is lower than the adult MTD. Weakly informative priors and all borrowing approaches perform very similar, only full borrowing has considerably worse accuracy.
15. $\alpha_{A}=\alpha_{P}$, $\beta_{A}<\beta_{P}$, ${\mathrm{MTD}}_{A}={\mathrm{MTD}}_{P}$. The intercepts are identical and the slopes are different. Pediatric MTD and adult MTD are identical. Correct information about the intercept outweighs the wrong information about the slope, so full borrowing performs better than the weakly informative priors. The models with estimated similarity parameter perform best, with 20 compounds even better than with 7 compounds, because compared to full borrowing they additionally discount the information on the slope and are therefore able to escalate faster.
16. $\alpha_{A}=\alpha_{P}$, $\beta_{A}=\beta_{P}$, ${\mathrm{MTD}}_{A}={\mathrm{MTD}}_{P}$. Intercepts and slopes are identical. The performance is as expected: full borrowing performs best, informed mixture and inflated priors come close, borrowing with default weights is somewhat worse, and weakly informative priors perform worst.

Overall, the information about other compounds could improve the accuracy of the dose‐finding in many scenarios compared to weakly informative priors and partial borrowing with default weights. The results were more consistent if 20 compounds were available than when only 7 compounds were available. The approach with mixture priors seemed to be more stable than the one with variance inflation, which in certain scenarios showed very poor performance. Differences between the ExNex and the hierarchical model used for similarity estimation were only marginal. In certain constellations, full borrowing performed surprisingly well, particularly if the strong prior information for the intercept indicated the correct MTD, which underlines the complexity of prior information for dose‐finding algorithms and the importance of careful investigation of all relevant scenarios in the planning stage of a study.

## 6 Case Study

In order to demonstrate an application of the proposed methods to estimate the similarity parameters, i.e., the first of the two parts of our simulation study, to real‐world data, we present a case study. We use data presented in Ollier et al. [17], who provided dose‐escalation data from 6 compounds (Eribulin, Lapatinib, Sorafenib, Ixabepilone, Edotecarin, e7070) in both Caucasian and Japanese patients. Although in the original publication the 6 compounds were analyzed separately from each other, we will treat them here as sufficiently similar to allow drawing conclusions about the similarity between Caucasian and Japanese patients in another biologically comparable compound that has been tested in European patients and is now to be tested in Japanese patients. A biological or medical analysis of the appropriateness of this assumption is outside of the scope of this case study, which is focused on the statistical aspects of the process.

The data is provided in Table 2 in Ollier et al. [17]. The number of dose levels varied between four and nine. Some doses were tested only in Caucasian patients and some only in Japanese patients. The sample sizes varied between 21 and 67 Caucasian patients and between 14 and 27 Japanese patients. We set the reference dose $d_{R}$ for each compound to be equal to the identified MTD, when this was the same for Japanese and Caucasian patients; for one compound, there was no MTD for Caucasian patients identified, so as reference dose, we specified the Japanese MTD; for another compound, the MTDs differed for both populations, and we defined the reference dose to be equal to the Caucasian MTD.

**FIGURE 4:**
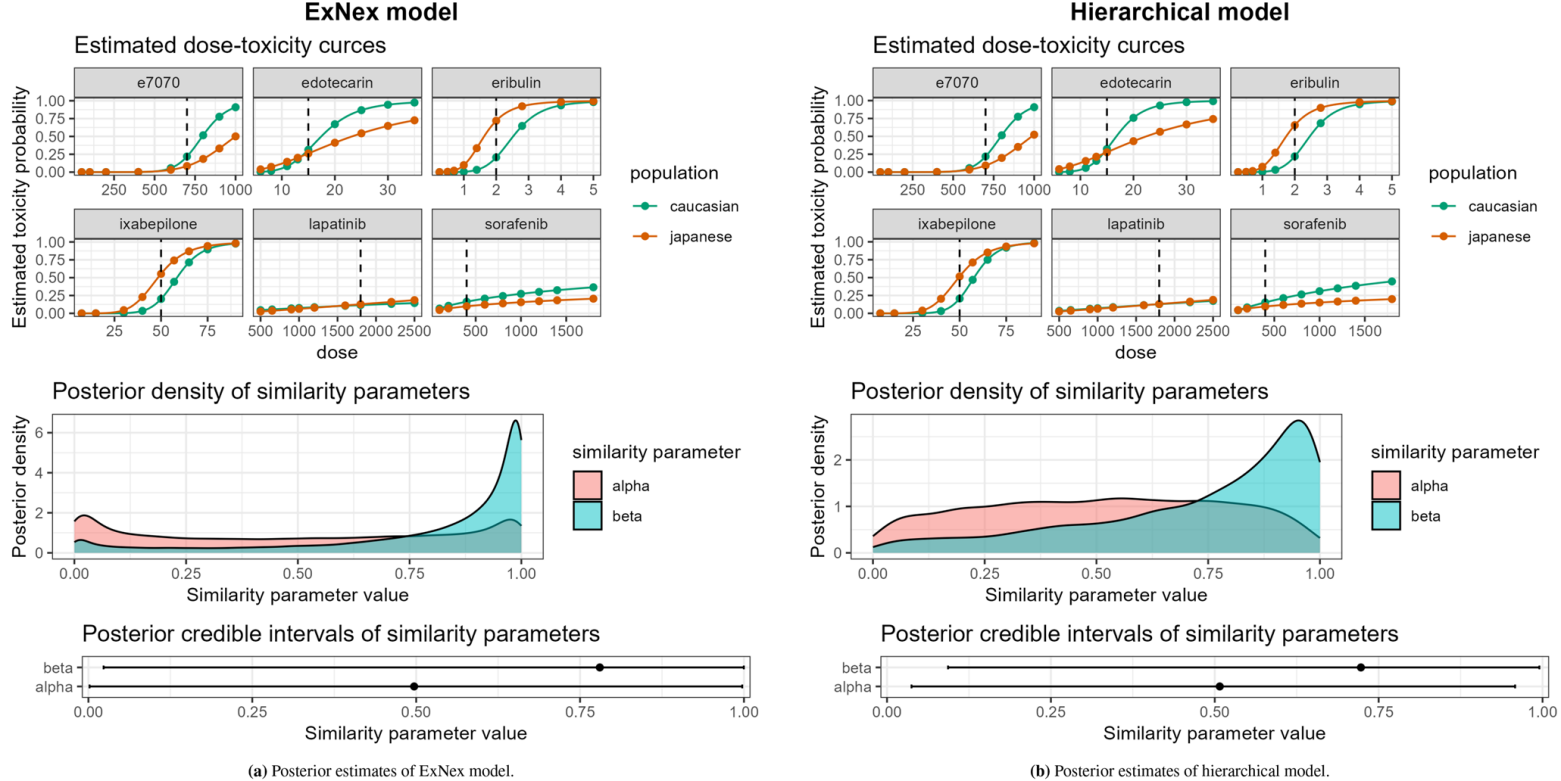
Case study: Comparing the results with the ExNex model and the hierarchical model. In the dose‐toxicity curves, the reference dose is indicated by the dashed line. In the 95% posterior credible intervals, the dot represents the posterior mean.

We fitted both the ExNex and the hierarchical model to this data. A summary of the results can be seen in Figure 4. Both models provided similar posterior estimation of the dose‐toxicity curves for each compound. Also, the posterior means of the similarity parameters were very close to each other. For the slope parameter, moderate similarity was assessed. The posterior means were 0.780 and 0.723, respectively, for the ExNex and the hierarchical model. About the similarity of the intercepts, no meaningful update of the prior information could be achieved (posterior means of 0.497 and 0.508, respectively), so there appears to be no clear evidence for either similar or non‐similar intercepts. This can be explained by the fact that four compounds were found to have similar intercepts, but two doses had very different intercepts. Hence, the models concluded that the intercepts were neither similar nor non‐similar. For demonstration purposes, we removed the two compounds with non‐similar intercepts and repeated the analysis. As a consequence, the estimated similarity of the intercepts increased. These results are shown in the Supporting Information.

Hence, the results of this analysis would support the recommendation of moderately increased weighting of information about the slope from data on European patients, and default weights with robust priors for the intercept.

## 7 Discussion

In this paper, we present methods for determining the degree of borrowing from a source population for a dose‐finding trial in the target population and investigate the potential benefit of their application using simulations and a case study.

Our approach consists of two separate information flows which are combined to inform the dose‐finding trial in the target population. First, the information from the preceding dose‐finding trial in the source population is expressed in an informative, robust prior distribution. We use a robust mixture distribution as described in Zocholl et al. [13] and variance inflation models similar to the ones suggested by Takeda and Morita [12]. Second, the similarity between dose‐toxicity curves from the source and target populations is estimated from other compounds. For this estimation, we present two methods, one using a hierarchical model and another applying an ExNex model. Both models estimate a similarity parameter for each of the two model parameters of the BLRM, which is applied to the dose‐finding trial in the target population. To combine both information flows, the estimated similarity parameter is transferred into the target population prior distribution and serves to control the degree of borrowing in the new dose‐finding trial.

To evaluate the performance of our approach under various circumstances, we implemented an extensive simulation study of several dose‐toxicity curves motivated by a pediatric‐adult setting. We considered different degrees of similarity between pediatric and adult dose‐toxicity curves and assumed situations from few to many available compounds to estimate this similarity. The simulation results show that both the ExNex model and the hierarchical approach enable reliable estimation of the similarity if there are many (i.e., 20) compounds available, and in that case the estimated similarity parameters can improve the pediatric dose‐finding across many toxicity scenarios compared to approaches with (weakly informative or informative) priors that do not consider this similarity information. Our approach appears to work more consistently with robust mixture priors than with variance inflation priors, which appear to be rather sensitive to the specific similarity parameter and require additional fine‐tuning, limiting the applicability of the method. The performance of our approach approximately reduces to that of default weighting when there are only 3 compounds available, because not enough data is available for a reliable estimation. With 7 compounds, the advantage over default weights is small, but notable. The performance of mixture priors with informed weighting based on 7 compounds is not in any of the 16 dose‐toxicity scenarios worse than that of the default weights. In several scenarios (to be specific: 6, 11, 12, 15, and 16), a moderate advantage is apparent, which is larger for weights based on ExNex than hierarchical modeling.

There remain some limitations to our study. First of all, we assumed that the new compound has the same similarity between target and source populations as the historical compounds. This may be seen as a strong assumption, and if it is violated obviously our approaches would not work as desired but instead would approach the performance of either weakly or strongly informative priors, whichever would be worse in the corresponding situation (simulation results under violation of this assumption are provided in the Supporting Information). But on the other hand, if one is not willing to make this assumption, it does not make sense to consider borrowing information from other compounds at all. For the sake of clarity and conciseness, our interpretation of the simulation results is based on the posterior means of the similarity parameters and the accuracy of the new pediatric trial. Other metrics could be of interest, too, such as the credible intervals of the similarity parameters and the number of DLTs or the allocation to the various doses in the pediatric trial. Further, to reliably estimate the dose‐toxicity curves of historical compounds, a certain number of DLTs need to be observed, which in practice may not always be the case due to drugs being very tolerable. Also, to what extent and in which situations the preceding trial in the source population serves as a good information source for the borrowing process is not investigated in this paper in detail. For an extensive discussion of this, we refer to previous simulation studies [13]. For the purpose of evaluating our approach here, we assume a moderately large adult trial with 40 adult patients tested with the same dosing scheme as the pediatric trial.

Although our approach aims to reduce subjectivity in the borrowing process, it is not entirely free from subjectivity. A particularly sensitive aspect is the selection of studies to be used for estimating the similarity parameters, which is demonstrated in the Supporting Information. In light of this, it should be noted that we do not propose a purely data‐driven approach to determine the borrowing weight. We acknowledge that there are other ways to determine the weight of the informative component, e.g., some general assessments by medical experts on the similarity between source and target populations, or setting a weight to achieve certain operational characteristics in a simulation study as suggested by one anonymous reviewer. However, we do think if such data is available from multiple external studies, then this information should be used in the most objective way possible. The different approaches to determine the degree of borrowing are not necessarily mutually exclusive but could also be combined. For example, the estimated similarity parameters could be critically assessed by medical experts and potentially adjusted accordingly. Additionally, the planning of a specific trial will always need to consider simulation‐based fine‐tuning of similarity parameters and prior distributions in order to avoid model‐based recommendations that are not aligned with medical judgment.

In this paper, we have proposed a novel approach to define an appropriate degree of borrowing in dose‐finding trials, which could particularly be used to borrow information between ethnicities or to inform a pediatric trial based on adult data. We have demonstrated how a similarity parameter that controls the degree of borrowing can be estimated from information from other compounds, and have shown via simulations that this can improve the accuracy of the pediatric dose‐finding trial. We have further presented a use case with borrowing from Caucasian to Japanese patients, illustrating the application with real‐world data. In practice, the decision whether to use the proposed approach will also be a cost‐related question: to gain a real advantage through estimating the similarity, information from many compounds is required, but if data is available without additional costs, it is probably worth investing time into the estimation of the similarity parameters. Our study confirms previous results [13] that even in the case of a lack of such data, and therefore poor estimation of the similarity parameters, robust priors are still likely to improve dose‐finding performance with little risk compared to weakly informative priors.

## Conflicts of Interest

The authors declare no conflicts of interest.

## Supporting information


**Data S1:** Supporting Information.

## Data Availability

The data that support the findings of this study are openly available in GitHub at https://github.com/dariozchl/Manuscript‐informative‐pediatric‐dose‐finding.
