# Supplementary material for: Informing the Borrowing Process for Dose‐Finding Trials by Estimating the Similarity Between Population‐Specific Dose‐Toxicity Curves
Source: Pharm Stat. 2025 Dec 19;25(1):e70067. doi: 10.1002/pst.70067 (PMC12742552; doi:10.1002/pst.70067)
Supplement: Supplementary file 1 — Data S1: Supporting Information. [file PST-25-0-s001.pdf]

Supplementary materials for
Informing the borrowing process for dose-finding trials by estimating the similarity between population-specific dose-toxicity curves

1 Choices of prior distributions

In the following, we describe the weakly informative priors that were used in the main paper. In Section 2.1, we describe that for the BLRM, $\sigma_\alpha = 2$,$\sigma_{\alpha,\beta} = 0$, and $\sigma_\beta = 1.5$ allow for a relatively wide range of toxicity probabilities for both the reference dose as well as a change of dose. In combination with prior means $\mu_\alpha = -0.84$ and $\mu_\beta = 0$, the median prior probability of toxicity for the reference dose is 0.289, with 20% and 80% quantiles being 0.073 and 0.691, respectively, allowing for the possibility of both severe underdosing as well as severe overdosing. Regarding the slope, a change of the dose from the reference dose to twice the amount results in an increase of the median toxicity probability to 0.466 with 20% and 80% quantiles being 0.346 and 0.843, respectively, representing both small and large changes of the toxicity probability.

For the estimation of similarity parameters, priors are defined for the ExNex (Section 3.1) and the hierarchical model (Section 3.2). For both models, all variance parameters are assigned Gamma priors, $\Gamma(1, 1/10)$, where we use the shape and inverse scale (or, rate) parameterization. Hence, the prior mean of each variance is 10, with 20% and 80% quantiles being 2.23 and 16.09, respectively, allowing for fairly large variances without being too diffuse. In the ExNex approach, each of the weights ζ_α and ζ_β, is assigned a Beta$(0.5, 0.5)$ distribution, which is a symmetrical U-shaped prior distribution with mean 0.5. In the hierarchical model, the correlation parameters ρ_τ and ρ_σ are assigned a uniform Unif$(-1, 1)$ distribution to allow for all possible values with equal probability.

2 Simulations with random scenarios

As pointed out by an anonymous reviewer, simulation scenarios do not need to be fixed but can also be generated randomly. For this, we assume that the parameters α and β, which are used to generate the toxicity data, are sampled from a normal distribution with means being equal to the fixed simulations scenarios (the *latent means*). We decided to keep pediatric scenarios to be not random, though, because if they would randomly differ from adult scenarios, it would be impossible to distinctly distinguish similar from non-similar scenarios. So, in our supplementary simulations, the α and β parameters for adult dose-toxicity scenarios are randomly generated (from a normal distribution with one randomly drawn mean from a set of two values, each), and pediatric dose-toxicity parameters are either the same or different. If they are different, pediatric parameters are drawn also from a normal distribution with mean other than the mean for adults. For the means of the normal distributions, we assumed the sets $\mu_\alpha \in \{-2.5, -0.84\}$ and $\mu_\beta \in \{0, 1.5\}$, and for the standard deviations we assumed $\sigma_{\alpha_A} = 0.3$ and $\sigma_{\beta_A} = 0.3$ for adult parameters and $\sigma_{\alpha_P} = 0.2$ and $\sigma_{\beta_P} = 0.2$ for pediatric parameters. The pediatric parameters were defined to vary less than the adult parameters to avoid that adults and pediatric dose-toxicity curves would not become too close just due to random deviation in scenarios with no true similarity. An overview of this data-generating mechanism is shown in Table 1.

Table 1: Overview of simulation data-generating mechanism.

**Aspect**	**Adults**	**Pediatrics**
Parameters	α_A, β_A	α_P, β_P
Distribution	$\alpha_A \sim N(\mu_{\alpha_A}, \sigma_{\alpha_A})$,<br>$\beta_A \sim N(\mu_{\beta_A}, \sigma_{\beta_A})$	in similar scenario:<br>$\alpha_A = \alpha_P, \beta_A = \beta_P$<br>in non-similar scenario:<br>$\alpha_P \sim N(\mu_{\alpha_P}, \sigma_{\alpha_P})$,<br>$\beta_P \sim N(\mu_{\beta_P}, \sigma_{\beta_P})$
Latent means	$\mu_\alpha \in \{-2.5, -0.84\}$ (randomly chosen)<br>$\mu_\beta \in \{0, 1.5\}$ (randomly chosen)	If non-similar scenario:<br>use the *other* mean from the sets left
Standard deviations	$\sigma_{\alpha_A} = 0.3$<br>$\sigma_{\beta_A} = 0.3$	$\sigma_{\alpha_P} = 0.2$<br>$\sigma_{\beta_P} = 0.2$
Scenario rules	Always random	Either identical to adult parameters<br>or sampled from Normal distribution
Rationale	Introduces random variability<br>in adult dose–toxicity curves	Smaller variability prevents accidental<br>overlap of curves in non-similar scenarios

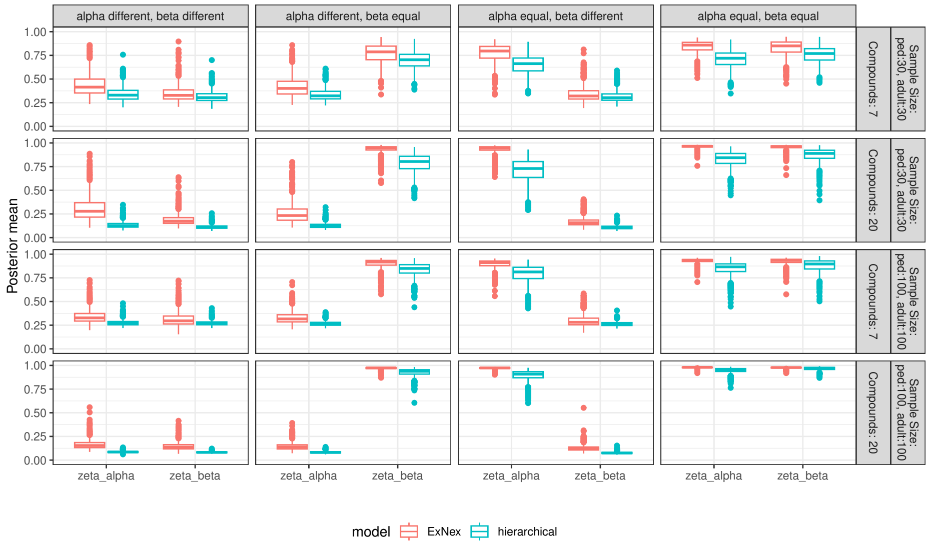

Figure 1: Visualization of simulation results for the estimation of similarity parameters based on 1000 simulations per scenario. Like Figure 2 in the main paper, but with random scenarios.

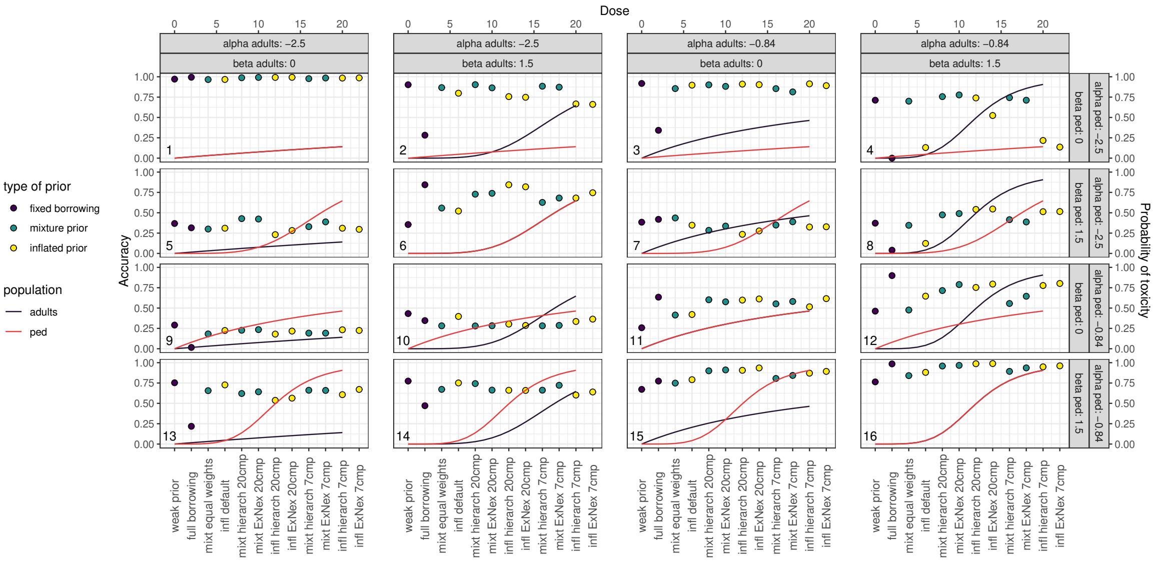

Figure 2: Visualization of simulation results for accuracy based on 1000 simulations per scenario. Like Figure 3 in the main paper, but with random scenarios.

The results with random scenarios very closely resemble the results from Figures 2 and 3 in the main paper.

3 Simulations with different starting dose

In the main paper, we have focused on "information borrowing" in terms of expressing the statistical information about the model parameters in the form of prior distributions. However, there other aspects about the dose-finding algorithm for which information can be borrowed, too. One of these is the starting dose of the pediatric trial. In the simulations in the main paper, we let all pediatric trials start at the first dose for safety reasons. However, one could also argue to start at the adult MTD, or one dose below the adult MTD, in order to avoid underdosing of the first cohorts of patients. As correctly noted by an anonymous reviewer, the starting dose, and the selection of doses in general, can significantly impact the operating characteristics and has been shown to be an important aspect of borrowing, particularly in strongly limited sample sizes (Zocholl et al. 2022). In order to demonstrate this, we have repeated the simulations of one selected scenario with particularly steep dose-toxicity curve (scenario 13, Figure 3).

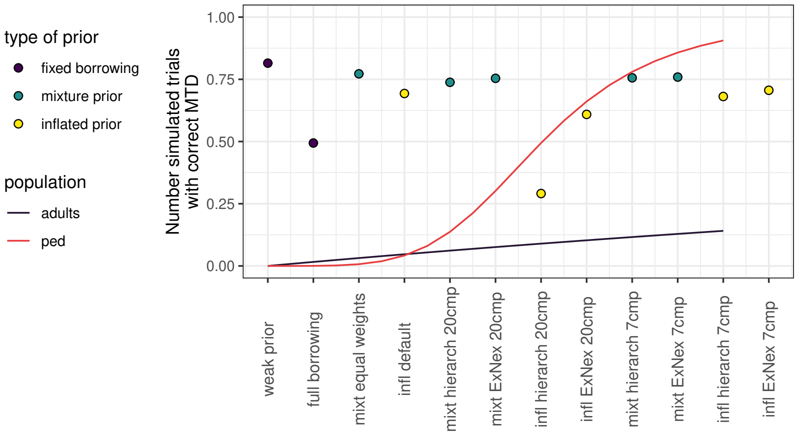

Figure 3: Visualization of results from 1,000 simulation runs for scenario 13 with starting dose of the pediatric trial being the adult MTD.

In this scenario, the pediatric dose-toxicity curve is much steeper than the adult dose-toxicity curve. The true pediatric MTD is dose level 3, while the true adult MTD is dose level 5. Therefore, if the starting dose of the pediatric trial is the adult MTD, the trials must first de-escalate. Compared to Figure 3 in the main paper, the accuracy for most algorithms is slightly increased, particularly because dose 4 is less often recommended because it is already identified as too toxic in the first few cohorts. When the trials are forced to start at the first dose, they cannot explore higher doses enough to correctly identify the high toxicity probability before the sample size of 12 pediatric patients is reached. However, in this specific scenario, starting at the adult MTD leads to more pediatric patients being overdosed (particularly in the first cohorts) and to a significant proportion of early termination, i.e., no dose is considered safe. Especially, the algorithms with inflated variance parameter suffer from this (in combination with weights estimated by the hierarchical model with 20 compounds, the proportion of early termination reached even 58%). This behavior can be controlled by adjusting the rules for early termination. Our dose-finding algorithms were calibrated for settings where the starting dose is the first dose and would terminate if the estimated toxicity probability of the first dose would be unacceptably toxic, i.e., above 40% ($\mathrm{E}(p_1) > 0.4$) – if a different starting dose is chosen, one would need to adjust the algorithm accordingly.

4 Simulations with wrong similarity estimates

In our simulations, we have assumed that the similarity between pediatric and adult patients is going to be the same for all compounds, in both historical and future trials. As a consequence, more historical data would always be better, as it improves estimation of the similarity parameters, which again improve operating characteristics of the future trial. However, one might wonder about the robustness against violations of this assumption. Consider the situation, where the relationship between adult and pediatric dose-toxicity curves of the new compound is different from that of the historical compounds used for the estimation of the similarity parameters. In order to address this, we have repeatedly simulated two scenarios: scenario 4, where both adult and pediatric parameters are different from each other but the estimation of similarity parameters was based on data from compounds, for which α and β parameters were equal; and scenario 11, where adult and pediatric parameters were the same but the estimation of similarity parameters was based on data from compounds, for which α and β parameters were different. The results are shown in Figure 4.

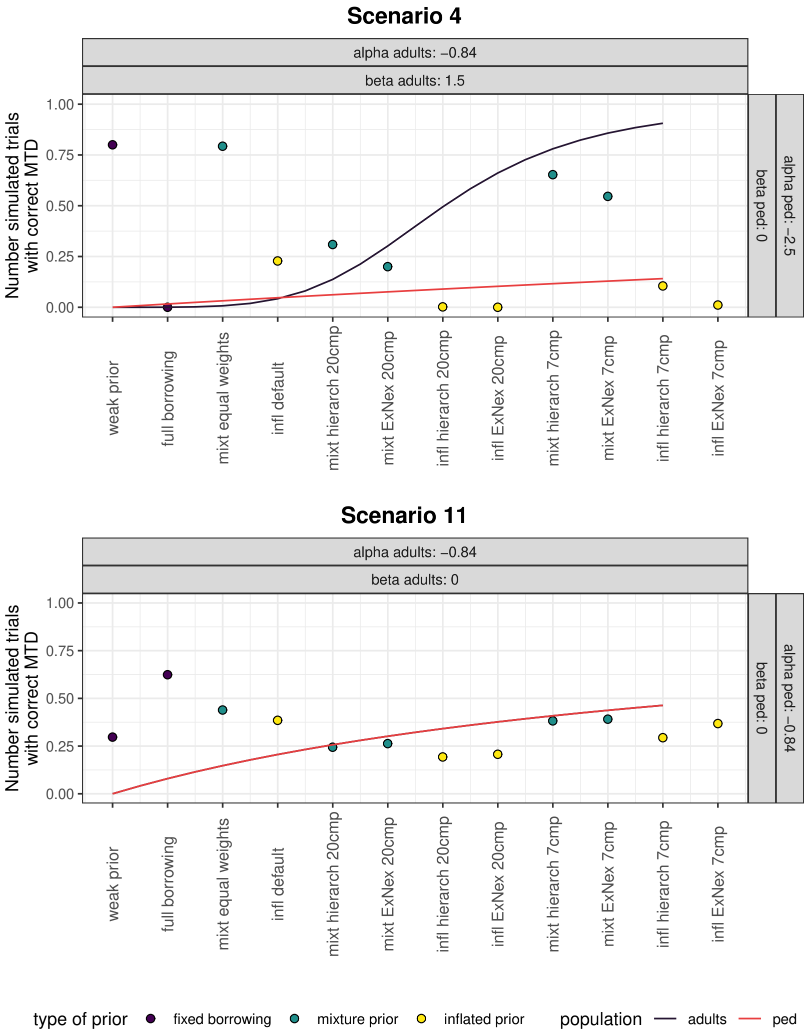

Figure 4: Visualization of results from 1,000 simulation runs for scenario 4 and scenario 11, where the estimation of the similarity parameters was based on the opposite scenario, i.e., for scenario 4, historical compounds had equal α and β parameters, and for scenario 11 they had different α and β parameters.

Compared to Figure 3 in the main paper, the results for the specifications, which do not use information about the estimated similarity parameters, are identical (up to random deviations). In contrast to this, the accuracy of the remaining configurations is affected, particularly the ones, where 20 compounds were used for estimating the similarity. In scenario 4, similarity parameters are falsely assumed to be large, therefore the accuracy with similarity parameters approaches the (extremely low) accuracy of full borrowing. For the models with inflated variance parameters, performance is particularly worse than with mixture priors, because they closely resemble full borrowing, while mixture priors still contain a non-informative component. With only 7 compounds, the mixture priors remain relatively robust and achieve still good accuracy.

In scenario 11, similarity parameters are falsely assumed to be close to 0, so models which use the information about the estimated similarity parameters approach the performance of weakly informative priors. The models with variance inflation and estimation based on 20 compounds show accuracy even slightly lower than that, which is due to strong variance inflation, resulting in overly diffuse priors. Again, based on 7 compounds, the mixture priors appear to be relatively robust against the violation of the assumption of equal similarity in historical and future compounds.

5 Case study: sensitivity analysis for selection of studies

Naturally, the estimation of the similarity parameter depends on the studies selected for the estimation. A sensitivity analysis can be useful in order to understand the impact of individual studies on the estimation. In the following, we have re-examined our case study by dropping two compounds from the analysis, for which the Caucasian and the Japanese MTD differed (Eribulin and Ixabepilone). As a consequence, the estimation based on the 4 remaining compounds yielded higher similarity for the intercept parameter (Figure 5), while the estimation of the similarity parameter for the slope parameter did not change much. The results were consistent with both the ExNex and the hierarchical model. This shows the sensitivity of the approach to the selection of the studies: while with all 6 compounds the estimation of the similarity of the intercepts was inconclusive, the information provided by the selected 4 compounds is consistent and the estimation supports borrowing of adult information about the intercept. Obviously, this sensitivity analysis is a toy example and in practice studies should never be selected or dropped based on their consistency with the remaining data.

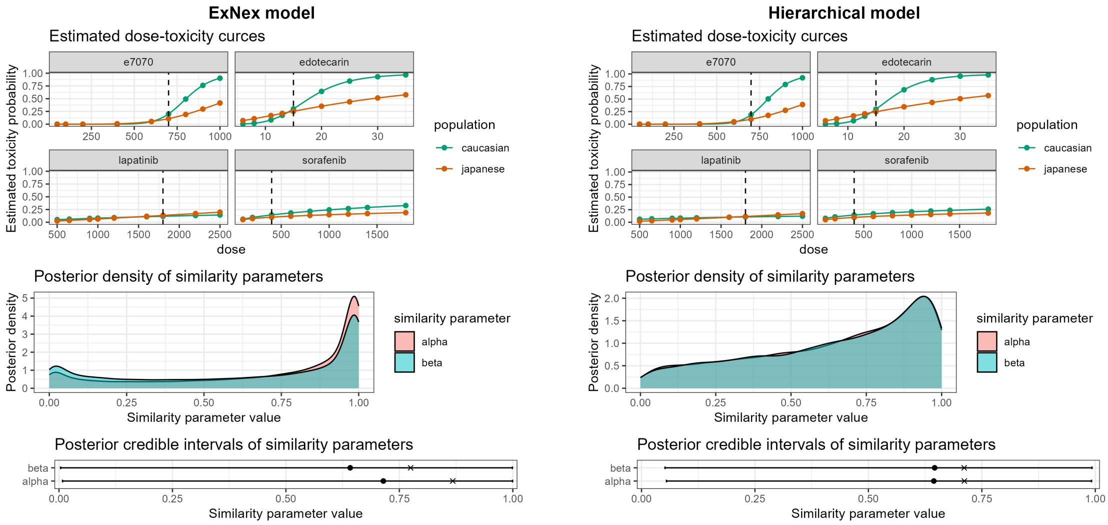

(a) Posterior estimates of ExNex model.

(b) Posterior estimates of hierarchical model.

Figure 5: Case study: comparing the results with the ExNex model with the hierarchical model. In the dose-toxicity curves, the reference dose is indicated by the dashed line. In the 95% posterior credible intervals, the dot represents the posterior mean, the x the posterior median.
